# An Energy Approach to the Modal Identification of a Variable Thickness Quartz Crystal Plate

**DOI:** 10.3390/s24206707

**Published:** 2024-10-18

**Authors:** Zhe Wang, Bin Huang, Yan Guo, Yanan Jiang, Asif Khan

**Affiliations:** 1 Zhejiang-Italy Joint Lab for Smart Materials and Advanced Structures, Faculty of Mechanical Engineering & Mechanics, Ningbo University, Ningbo 315211, China; 2 College of Science & Technology, Ningbo University, Ningbo 315300, China; 3 Mechanical Engineering Program, Physical Science and Engineering Division, King Abdullah University of Science and Technology (KAUST), Thuwal 239556900, Saudi Arabia

**Keywords:** modal identification, energy method, thickness–shear vibration, variable thickness, beveling

## Abstract

The primary objective of modal identification for variable thickness quartz plates is to ascertain their dominant operating mode, which is essential for examining the vibration of beveled quartz resonators. These beveled resonators are plate structures with varying thicknesses. While the beveling process mitigates some spurious modes, it still presents challenges for modal identification. In this work, we introduce a modal identification technique based on the energy method. When a plate with variable thickness is in a resonant state of thickness–shear vibration, the proportions of strain energy and kinetic energy associated with the thickness–shear mode in the total energy reach their peak values. Near this frequency, their proportions are the highest, aiding in identifying the dominant mode. Our research was based on the Mindlin plate theory, and appropriate modal truncation were conducted by retaining three modes for the coupled vibration analysis. The governing equation of the coupled vibration was solved for eigenvalue problem, and the modal energy proportions were calculated based on the determined modal displacement and frequency. Finally, we computed the eigenvalue problems at different beveling time, as well as the modal energies associated with each mode. By calculating the energy proportions, we could clearly identify the dominant mode at each frequency. Our proposed method can effectively assist engineers in identifying vibration modes, facilitating the design and optimization of variable thickness quartz resonators for sensing applications.

## 1. Introduction

Modal identification is crucial in engineering and scientific fields, particularly in structural engineering, mechanical engineering, aerospace engineering, etc. Its primary functions include assessing structural health status, fault diagnosis, noise and vibration control, etc. [1,2]. However, the development of modal identification technology faces numerous challenges, such as noise interference, data acquisition and processing, accuracy of modal parameter identification, nonlinear and time-varying characteristics, and the multimodal characteristics of complex structures. This is especially true for structures with multiple degrees of freedom, non-uniform material distribution, or anisotropic material properties, where modal characteristics can be highly complex and the coupling between modes further complicates identification. Quartz crystal resonators (QCR), made from piezoelectric quartz material and usually in rectangular or circular plate forms, are frequency control components widely used in electronic devices [3,4]. They provide stable clock signals to ensure the synchronization and accuracy of data transmission and are extensively used in electronic devices. They are also widely used as filters, force-frequency sensors, microbalances, and temperature sensors. Due to the thinness of the quartz wafer and the anisotropic nature of the material, the operating mode excited in quartz plate vibrations is highly complex, involving the coupling of multiple vibration modes rather than a “clean” single mode [5]. This mode interference affects the performance of resonators. Therefore, accurately identifying the operating mode and guiding the design of resonators is essential for the development of resonator technology.

In the field of resonator design, the operating mode of the device is generally referred to as the main mode, and other modes that interfere with the main mode are referred to as spurious modes [6,7]. In quartz crystal resonators, thickness–shear mode (TSH) is one of the main operating modes, but there are multiple spurious modes around the operating frequency. The TSH mode is harmonic and antisymmetric through the thickness of plate. At present, quartz crystal resonators are developing towards smaller sizes and higher frequencies. The smaller the chip size is, the more likely it is to produce more severe spurious mode and operating mode coupling phenomena, which is currently one of the main technical difficulties in resonator design [8,9]. Due to the significant impact of spurious modes on the performance of resonators, not only should the dominant operating mode be determined in the design of resonators, but also the spurious modes near the operating mode should be found. Therefore, due to the coupling between spurious modes and operating modes, it is crucial to identify all modes and further use them to design and eliminate the influence of spurious modes.

In order to suppress or eliminate the influence of spurious modes, adjusting the morphology of quartz crystal blank and designing proper shape of electrodes are two of the main methodologies [10,11]. In production, quartz blanks are often subjected to beveling treatment. Quartz blanks are usually processed into a shape with a thick center and thin sides, which are called beveled resonators [12]. The beveling process involves fixing sandpaper on the inside of the drum, placing the quartz crystal blank into the drum, and then gradually grinding off the edges and corners of the blank through rotation to achieve the purpose of beveling. The final morphology of the blank formed by grinding is related to the curvature of the drum, the size of the sand particles, and the parameters of the beveling process. The beveling process can suppress the vibration of the plate in its central region. For quartz resonators, when the sound waves excited by the electrode propagate out of the electrode region, their energy rapidly decays, causing almost all of the vibration energy to be concentrated in the central electrode region, and this phenomenon is called the energy trapping effect [13,14]. This approach can not only reduce the impact of edge clamping but also suppress spurious modes. In design, selecting the optimal aspect ratio can also achieve a relatively ‘clean’ thickness–shear mode, thereby reducing coupling with other modes. Meanwhile, the treatment of electrode shape and position is also an important method to suppress spurious modes, which enhances the energy trapping effect to suppress the influence of spurious modes. The energy trapping effect can effectively suppress vibration energy in the working area and effectively suppress spurious modes. This characteristic helps to improve the reliability and stability of quartz resonators.

Until now, a large number of works has been carried out for the analysis of coupled vibration and energy trapping effect of quartz crystals with non-uniform thickness. Understanding the vibration behavior is a prerequisite for designing resonant sensors. Mindlin and Forray proposed an approximate method for calculating the frequencies of thickness–shear vibration and flexural vibration of variable thickness plates and applied it to plates having the shape of a double wedge and to plates with beveled edges [15]. Yang and Batra investigated the thickness–shear vibrations of a circular cylindrical piezoelectric shell analytically [16]. Wang and Lee et al. calculated the modal shapes and frequency spectra for various parameters of strip resonators with beveled edges and discussed the influence of the contouring [17]. Slavov introduced an equivalent resonance radius into AT-cut quartz crystals based on the coupled vibration theory and studied the relationship between motion inductance and postmetallization resonance frequency change, electrode dimensions and spherical contour radius of piezoelectric elements through experimental investigations [18]. Tiersten et al. also applied the equation for transversely varying thickness modes in doubly rotated quartz resonators for the analysis of contoured resonators with rectangular electrodes [19]. The influence of both contouring and continuity conditions at the edges of the electrodes were included in the analysis. Many of the theoretical works of beveled resonators have been carried out based on the scalar differential equations derived by Tiersten for AT-cut and SC-cut quartz resonators [20]. Li conducted vibration analyses of thickness–shear vibration of AT-cut quartz resonator with hyperbolic thickness variation based on the Legendre equation and hypergeometric function [21]. Subsequently, he used the power series expansion technique to establish a vibration model for a contoured quartz crystal plate with continuous thickness variation [22]. Wang et al. analyzed the free vibration of an elliptical AT-cut crystal resonator with quadratic thickness variation based on the scalar equation [23]. In addition to the aforementioned geometric models, there are other types of thickness variation models such as cubic [24] or step functions [25,26].

The modal identification referred to in this article mainly focuses on identifying the dominant operating mode of beveled quartz resonators. The vibration modes in finite quartz crystal plates are coupled and complex. To avoid strong coupling between the operating mode and other spurious modes, identification of the operating mode is very important. Furthermore, due to the anisotropic material properties of quartz, the vibration modes are more complex and difficult to accurately identify. Conventionally, modal identification is accomplished by drawing modal diagrams where the normalized vibration modes are drawn on the same graph, and then the dominant mode is identified by manually analyzing the magnitudes of all modes. Yong et al. studied the vibration modes of quartz plates based on Mindlin plate theory [27] and identified the vibration modes through modal diagrams. Wang et al. also conducted modal analysis of AT-cut quartz resonators [28]. In addition, some scholars have used the energy method to identify and analyze the vibration modes of structures. The so-called energy method is to determine the proportion of elastic potential energy and kinetic energy of each mode in the total energy at different frequencies. Huang et al. analyzed quartz plate vibration with multiple modes using the energy method but without considering the coupling energy [29]. When vibration analysis requires consideration of higher orders and more modes, manually identifying these modes will become extremely complex. Using the energy method to automatically determine the dominant mode ensures high identification accuracy and greatly improves computational efficiency. Therefore, this method has important application value in the field of coupled vibration and can be used as an effective tool for modal identification of multimode coupled vibration.

In this work, we mainly propose to use the energy method to study the vibration mode identification of beveled resonators. Our work will focus on the modeling of multimode coupled vibration of variable thickness plates based on Mindlin plate theory with three modes, as well as solving free vibration problems using numerical analysis package. For the variable thickness plate model, this study will use the polynomial geometric model determined by the morphology data measured during the beveling process. This will accurately estimate the geometric morphology of the quartz plate after beveling and make precise predictions for both dominant and spurious modes. Finally, the strain energy and kinetic energy expressions of different modes are given, and the energy proportion of each mode in one vibration period at each frequency is calculated to help identify the dominant mode. The principle of this method is simple, the calculation is convenient, and it is not limited by the structural shape. It can accurately identify the dominant mode at different frequencies and provide an effective tool for identifying complex modes of beveled quartz crystals.

## 2. Mathematical Model

### 2.1. Basic Equations of Mindlin Plate Theory

The mechanical model for variable thickness piezoelectric quartz plates is derived from a three-dimensional linear elastic model. The energy density function of the piezoelectric medium considering the electromechanical coupling energy is given in the following equation.

(1) $$H(S_{ij},E_{j})=\frac{1}{2}c_{ijkl}S_{ij}S_{kl}-e_{kij}S_{ij}E_{k}-\frac{1}{2}\varepsilon_{ij}E_{i}E_{j}$$

where *E_k_* is the electric field tensor, *S_ij_* is the strain tensor, *c_ijkl_* is the elastic constant, *e_ijk_* is the piezoelectric constant, and *ε_ij_* is the dielectric constant.

From the above equation, the constitutive relationship can be further obtained and shown in the following equation.

(2) $$T_{ij}=\frac{\partial H}{\partial S_{ij}}=c_{ijkl}S_{kl}-e_{kij}E_{k}\;D_{i}=-\frac{\partial H}{\partial E_{i}}=e_{ijk}S_{jk}+\varepsilon_{ij}E_{j}$$

where *T_ij_* is the stress tensor, and *D_i_* is the electric displacement.

In linear elastic theory, considering small deformation, we can directly give the following geometric equations and electric potential equations.

(3) $$\begin{array}{c} S_{ij}=\left(u_{i,j}+u_{j,i}\right)/2\;\\ E_{i}=-\varphi_{,i} \end{array}$$

where *u_i_* represents the displacement, *φ* represents the potential, and subscript (, *i*) represents the partial differential of *i*.

Consider the following variational equation:

(4) $$\begin{array}{cc} \delta \Pi \left(u,\varphi \right) & =\int_{0}^{t_{0}}dt\int_{V}\left[\left(T_{ij,j}-\rho {\ddot{u}}_{i}+\rho f_{i}\right)\delta u_{i}+\left(D_{i,i}-\rho_{e}\right)\delta \varphi \right]dV\\ & +\int_{0}^{t_{0}}dt\int_{A}\left({\bar{t}}_{i}-T_{ij}n_{j}\right)\delta u_{i}dA-\int_{0}^{t_{0}}dt\int_{A}\left({\bar{\sigma }}_{e}+D_{i}n_{i}\right)\delta \varphi dA \end{array}$$

where *ρ* represents the density, *f_i_* represents the body force, *ρ_e_* represents the free charge density per unit reference volume, ${\bar{t}}_{i}$ represents the prescribed traction, ${\bar{\sigma }}_{e}$ represents the free charge density per unit surface aera, *n_i_* and *n_j_* are the unit outward normals.

**Figure 1:**
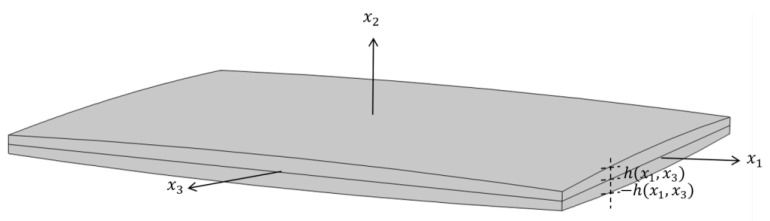
Geometry and coordinate system of a rectangular plate with variable thickness.

Therefore, the governing equations and natural boundary conditions can be obtained from the stationary condition of the above variation. For the present problem, Figure 1 shows the geometric model and coordinates of the vertically symmetrical variable thickness plate used in this work. The variable *x*_2_ is the thickness direction, and the thickness is a function of coordinates. Therefore, *h*(*x*_1_, *x*_3_) can be set as the morphology function of the half thickness of the quartz plate. According to the Mindlin plate theory, the displacement *u_i_* and electric potential *φ* can be expanded into the following series form [30].

(5) $$\begin{array}{l} u_{i}\left(x_{1,}x_{2,}x_{3,}t\right)=\sum_{n=0}^{\infty }u_{i}^{\left(n\right)}\left(x_{1},x_{3},t\right)x_{2}^{n}\\ \varphi \left(x_{1,}x_{2,}x_{3,}t\right)=\sum_{n=0}^{\infty }\varphi^{\left(n\right)}\left(x_{1},x_{3},t\right)x_{2}^{n}\left(i=1,2,3\right) \end{array}$$

where $u_{i}^{\left(n\right)}$ and $\varphi^{\left(n\right)}$ are the *n*th order displacement component and potential component, which are only related to coordinates *x*_1_ and *x*_3_, as well as time *t*, and are not related to *x*_2_.

By substituting the displacement equation in power series form into the geometric equation, electric potential equation, and constitutive relationship, the high-order strain, high-order electric field, and high-order constitutive equation can be obtained. Thus, the high-order strain can be expressed in Equation (6).

(6) $$S_{ij}^{\left(n\right)}=\frac{1}{2}\left[u_{i,j}^{\left(n\right)}+u_{j,i}^{\left(n\right)}\right.+(n+1)\left.\left(\delta_{2j}u_{i}^{\left(n+1\right)}+\delta_{2i}u_{j}^{\left(n+1\right)}\right)\right]$$

where

$$\delta_{2j}=\left\{\begin{array}{c} 1,\;j=2\\ 0,\;j\neq 2 \end{array}\right.$$

And the *n*th order electric field can be expressed in Equation (7).

(7) $$E_{i}^{\left(n\right)}=-\varphi_{,i}^{\left(n\right)}-(n+1)\delta_{i2}\varphi^{\left(n+1\right)}$$

And the *n*th order constitutive equation can also be expressed in Equation (8).

(8) $$\begin{array}{c} T_{ij}^{\left(n\right)}=\sum_{m=0}B_{mn}(c_{ijkl}S_{kl}^{\left(m\right)}-e_{kij}E_{k}^{\left(m\right)})\\ D_{i}^{\left(n\right)}=\sum_{m=0}B_{mn}(e_{ijk}S_{jk}^{\left(m\right)}+\varepsilon_{ij}E_{j}^{\left(m\right)}) \end{array}$$

where

$$\begin{array}{c} T_{ij}^{\left(n\right)}=\int_{-h}^{h}T_{ij}x_{2}^{n}dx_{2},\;D_{i}^{\left(n\right)}=\int_{-h}^{h}D_{i}x_{2}^{n}dx_{2}\\ B_{mn}=\left\{\begin{array}{c} \frac{2h^{m+n+1}}{m+n+1},\;m+n=even\\ 0\;,\;m+n=odd \end{array}\right. \end{array}$$

By substituting the above equation into the governing equation, Equation (4), the governing equation for the two-dimensional Mindlin plate theory can be obtained as follows.

(9) $$\begin{array}{c} T_{ij,i}^{\left(n\right)}-nT_{2j}^{\left(n-1\right)}+F_{j}^{\left(n\right)}=\rho \sum_{m}B_{mn}{\ddot{u}}_{j}^{\left(m\right)}\\ D_{i,i}^{\left(n\right)}-nD_{2}^{\left(n-1\right)}+B_{mn}D^{\left(n\right)}=0 \end{array}$$

where

$$D^{\left(n\right)}={\left[D_{2}x_{2}^{n}\right]}_{-h}^{h},\;\;\;F_{j}^{\left(n\right)}={\left[T_{2j}x_{2}^{n}\right]}_{-h}^{h}$$

By substituting the high-order strain, Equation (6), into the high-order constitutive equation, Equation (8), the detail expressions of the high-order constitutive equation can be obtained, which are expressed as follows.

(10) $$\begin{array}{l} \begin{array}{l} T_{ij}^{\left(n\right)}=\sum_{m=0}^{\infty }B_{mn}\left\{c_{p1}u_{1,1}^{\left(m\right)}+\right.c_{p2}(m+1)u_{2}^{\left(m+1\right)}+c_{p4}\left[u_{2,3}^{\left(m\right)}+(m+1)u_{3}^{\left(m+1\right)}\right]\\ \;{+c}_{p3}u_{3,3}^{\left(m\right)}+c_{p5}(u_{3,1}^{\left(m\right)}+u_{1,3}^{\left(m\right)}+c_{p6}\left[u_{2,1}^{\left(m\right)}+(m+1)u_{1}^{\left(m+1\right)}\right]\\ \;+e_{1p}\varphi_{,1}^{\left(m\right)}+e_{2p}(m+1)\varphi^{\left(m+1\right)}\left.+e_{3p}\varphi_{,3}^{\left(m\right)}\right\} \end{array}\\ \begin{array}{l} D_{i}^{\left(n\right)}=\sum_{m=0}^{\infty }B_{mn}\left\{e_{i1}u_{1,1}^{\left(m\right)}+\right.e_{i2}(m+1)u_{2}^{\left(m+1\right)}+e_{i3}u_{3,3}^{\left(m\right)}+e_{i4}\left[u_{2,3}^{\left(m\right)}+(m+1)u_{3}^{\left(m+1\right)}\right]\\ \;{+e}_{i5}\left(u_{3,1}^{\left(m\right)}+u_{1,3}^{\left(m\right)}\right)+e_{i6}\left[u_{2,1}^{\left(m\right)}+(m+1)u_{1}^{\left(m+1\right)}\right]\\ \;-\varepsilon_{i1}\varphi_{,1}^{\left(m\right)}-\varepsilon_{i2}k_{2}^{\left(m\right)}(m+1)\varphi^{\left(m+1\right)}\left.+\varepsilon_{i3}\varphi_{,3}^{\left(m\right)}\right\} \end{array} \end{array}$$

### 2.2. Formulations Based on Modal Truncation

In practice, the coupling effect of the first-order thickness–shear mode (TSH), flexural mode (F), and face–shear mode (FS) of the plate is severe. Therefore, we considered the coupling vibration between these three modes, reflected in the simplification of the Mindlin equation, which greatly simplified the calculation process. Therefore, the displacement equation becomes:

(11) $$u_{1}=u_{1}^{\left(1\right)}x_{2},\;\;\;\;u_{2}=u_{2}^{\left(0\right)},\;\;\;\;u_{3}=u_{3}^{\left(0\right)}$$

where $u_{1}^{\left(1\right)}$ is the first-order thickness–shear mode, $u_{2}^{\left(0\right)}$ is the flexural mode, and $u_{3}^{\left(0\right)}$ is the face–shear mode.

By substituting Equation (11) into the high-order strain Equation (6), non-zero zeroth and first-order strain equations can be obtained and expressed in the following equation.

(12) $$\begin{array}{c} S_{3}^{\left(0\right)}=\frac{\partial u_{3}^{\left(0\right)}}{\partial x_{3}},\;\;\;S_{4}^{\left(0\right)}=\frac{\partial u_{2}^{\left(0\right)}}{\partial x_{3}},\;\;\;S_{5}^{\left(0\right)}=\frac{\partial u_{3}^{\left(0\right)}}{\partial x_{1}}+\frac{\partial u_{1}^{\left(0\right)}}{\partial x_{3}},\;\;\;S_{6}^{\left(0\right)}=\frac{\partial u_{2}^{\left(0\right)}}{\partial x_{1}}+u_{1}^{\left(1\right)}\\ S_{1}^{\left(1\right)}=\frac{\partial u_{1}^{\left(1\right)}}{\partial x_{1}},\;\;S_{5}^{\left(1\right)}=\frac{\partial u_{1}^{\left(1\right)}}{\partial x_{3}} \end{array}$$

By ignoring the piezoelectric effect, the first equation in Equation (9) becomes the following form.

(13) $$\begin{array}{c} \frac{\partial T_{6}^{\left(0\right)}}{\partial x_{1}}+\frac{\partial T_{4}^{\left(0\right)}}{\partial x_{3}}=2h\rho {\ddot{u}}_{2}^{\left(0\right)}\\ \frac{\partial T_{5}^{\left(0\right)}}{\partial x_{1}}+\frac{\partial T_{3}^{\left(0\right)}}{\partial x_{3}}=2h\rho {\ddot{u}}_{3}^{\left(0\right)}\\ \frac{\partial T_{1}^{\left(1\right)}}{\partial x_{1}}+\frac{\partial T_{5}^{\left(1\right)}}{\partial x_{3}}-T_{6}^{\left(0\right)}=\frac{2}{3}h^{3}\rho {\ddot{u}}_{1}^{\left(1\right)} \end{array}$$

Considering the AT-cut quartz plate, the material stiffness matrix can be found in the reference [30]. Using the stiffness matrix, the zero-order stress component used in the above equation can be simplified as

(14) $$\begin{array}{c} T_{3}^{\left(0\right)}=2h(c_{33}\frac{\partial u_{3}^{\left(0\right)}}{\partial x_{3}}+c_{34}\frac{\partial u_{2}^{\left(0\right)}}{\partial x_{3}})\\ T_{4}^{\left(0\right)}=2h(c_{43}\frac{\partial u_{3}^{\left(0\right)}}{\partial x_{3}}+c_{44}\frac{\partial u_{2}^{\left(0\right)}}{\partial x_{3}})\\ T_{5}^{\left(0\right)}=2h(c_{55}\frac{\partial u_{3}^{\left(0\right)}}{\partial x_{1}}+c_{56}\frac{\partial u_{2}^{\left(0\right)}}{\partial x_{1}}+c_{66}u_{1}^{\left(1\right)})\\ T_{6}^{\left(0\right)}=2h(c_{65}\frac{\partial u_{3}^{\left(0\right)}}{\partial x_{1}}+c_{66}\frac{\partial u_{2}^{\left(0\right)}}{\partial x_{1}}+c_{66}u_{1}^{\left(1\right)}) \end{array}$$

And the first-order stress component is simplified as

(15) $$T_{1}^{\left(1\right)}=\frac{2h^{3}}{3}c_{11}\frac{\partial u_{1}^{\left(1\right)}}{\partial x_{1}},\;\;\;T_{5}^{\left(1\right)}=\frac{2h^{3}}{3}c_{55}\frac{\partial u_{1}^{\left(1\right)}}{\partial x_{3}}$$

It was found that the right side of the governing equation, Equation (13), included the variable thickness value *h*, and its solution was difficult to solve analytically for the three-mode coupled vibration. Therefore, we adopted the numerical solution method to calculate the eigenvalue problem of the governing equation. We used the partial differential equation module provided by COMSOL to solve the coupled vibration. By rewriting the governing equation according to the definition form of the partial differential equation module and inputting it into the software for eigenvalue solving, we could obtain the vibration frequencies and modal solutions of the coupled vibration. We further utilized the free vibration solutions for modal identification in this work.

### 2.3. Energy-Based Modal Identification Method

The energy based modal identification method distinguishes each mode based on the proportion of strain energy or kinetic energy of each mode in all modal energies. Thus, it is necessary to calculate the strain energy and kinetic energy of each mode. Firstly, we calculated the proportion of strain energy for each mode. According to Mindlin plate theory, the expression for first-order strain energy density can be written as the following equation [30].

(16) $$\bar{U}=\frac{1}{2}c_{pq}\sum_{m}\sum_{n}B_{mn}S_{p}^{\left(m\right)}S_{q}^{\left(n\right)}=\frac{1}{2}(B_{00}S_{p}^{\left(0\right)}S_{q}^{\left(0\right)}+B_{11}S_{p}^{\left(1\right)}S_{q}^{\left(1\right)})$$

Expanding the above equation yields the following equation.

(17) $$\begin{array}{c} \bar{U}=\frac{1}{2}B_{00}c_{33}S_{3}^{\left(0\right)}S_{3}^{\left(0\right)}+\frac{1}{2}B_{00}c_{44}S_{4}^{\left(0\right)}S_{4}^{\left(0\right)}+\frac{1}{2}B_{00}c_{55}S_{5}^{\left(0\right)}S_{5}^{\left(0\right)}+\frac{1}{2}B_{00}c_{66}S_{6}^{\left(0\right)}S_{6}^{\left(0\right)}\\ +B_{00}c_{34}S_{3}^{\left(0\right)}S_{4}^{\left(0\right)}+B_{00}c_{56}S_{5}^{\left(0\right)}S_{6}^{\left(0\right)}+\frac{1}{2}B_{11}c_{11}S_{1}^{\left(1\right)}S_{1}^{\left(1\right)}+\frac{1}{2}B_{11}c_{55}S_{5}^{\left(1\right)}S_{5}^{\left(1\right)} \end{array}$$

Substituting the strain displacement relationship, Equation (12) into Equation (17), the strain energy density function becomes the following form.

(18) $$\begin{array}{cc} \bar{U} & =\frac{1}{2}B_{00}c_{44}{\left(u_{2,3}^{\left(0\right)}\right)}^{2}+\frac{1}{2}B_{00}c_{66}{\left(u_{2,1}^{\left(0\right)}\right)}^{2}+\frac{1}{2}B_{00}c_{55}{\left(u_{3,1}^{\left(0\right)}\right)}^{2}+\frac{1}{2}B_{00}c_{33}{\left(u_{3,3}^{\left(0\right)}\right)}^{2}\\ & +\frac{1}{2}B_{00}c_{66}{\left(u_{1}^{\left(1\right)}\right)}^{2}+\frac{1}{2}B_{11}c_{11}{\left(u_{1,1}^{\left(1\right)}\right)}^{2}+\frac{1}{2}B_{11}c_{55}{\left(u_{1,3}^{\left(1\right)}\right)}^{2}\\ & +B_{00}c_{66}u_{2,1}^{\left(0\right)}u_{1}^{\left(1\right)}+B_{00}c_{56}u_{2,1}^{\left(0\right)}u_{3,1}^{\left(0\right)}+B_{00}c_{34}u_{2,3}^{\left(0\right)}u_{3,3}^{\left(0\right)}+B_{00}c_{56}u_{3,1}^{\left(0\right)}u_{1}^{\left(1\right)} \end{array}$$

Next, we classified the energy and provide the strain energy and coupling energy densities between each mode, as shown in Equation (19) below.

(19) $$\begin{array}{c} {\bar{U}}_{TSH}=\frac{1}{2}B_{00}c_{66}{\left(u_{1}^{\left(1\right)}\right)}^{2}+\frac{1}{2}B_{11}c_{11}{\left(u_{1,1}^{\left(1\right)}\right)}^{2}+\frac{1}{2}B_{11}c_{55}{\left(u_{1,3}^{\left(1\right)}\right)}^{2}\\ {\bar{U}}_{F}=\frac{1}{2}B_{00}c_{66}{\left(u_{2,1}^{\left(0\right)}\right)}^{2}+\frac{1}{2}B_{00}c_{44}{\left(u_{2,3}^{\left(0\right)}\right)}^{2}\\ {\bar{U}}_{FS}=\frac{1}{2}B_{00}c_{55}{\left(u_{3,1}^{\left(0\right)}\right)}^{2}+\frac{1}{2}B_{00}c_{33}{\left(u_{3,3}^{\left(0\right)}\right)}^{2}\\ {\bar{U}}_{F-TSH}=B_{00}c_{66}u_{2,1}^{\left(0\right)}u_{1}^{\left(1\right)}\\ {\bar{U}}_{F-FS}=B_{00}c_{56}u_{2,1}^{\left(0\right)}u_{3,1}^{\left(0\right)}+B_{00}c_{34}u_{2,3}^{\left(0\right)}u_{3,3}^{\left(0\right)}\\ {\bar{U}}_{FS-TSH}=B_{00}c_{56}u_{3,1}^{\left(0\right)}u_{1}^{\left(1\right)} \end{array}$$

where ${\bar{U}}_{TSH}$, ${\bar{U}}_{F}$, and ${\bar{U}}_{FS}$ are the strain energy densities of thickness–shear mode, flexural mode, and face–shear mode, respectively. ${\bar{U}}_{F-TSH}$, ${\bar{U}}_{F-FS}$, and ${\bar{U}}_{FS-TSH}$ are the coupling energy between different modes. It is worth noting that the strain energy densities given above are based on the stiffness parameters of AT-cut quartz. The expression for strain energy densities needs to be adjusted based on the stiffness matrix for quartz materials with different cutting types.

The strain energy of a variable thickness plate can be obtained by integrating the energy density within one vibration period T, which is expressed in the following equation.

(20) $$U_{N}=\int_{-a}^{a}\int_{-c}^{c}\int_{0}^{T}{\bar{U}}_{N}dx_{1}dx_{3}dt,\;\;\;\;\;\left(N=TSH,FS,F,F-TSH,TSH-FS\right)$$

The total strain energy of all modes within one vibration period can be written as the following equation.

(21) $$U_{T}=\sum U_{N}=U_{TSH}+U_{F}+U_{FS}+U_{F-TSH}+U_{F-FS}+U_{TSH-FS}$$

In previous numerical analysis, we have already solved for the modal analysis. And now in post-processing, we could use the energy formula, Equation (19), to calculate the strain energies of all modes. The energy proportion of each mode is given below to achieve the identification of the operating mode as shown in Equation (22).

(22) $$\begin{array}{l} P_{TSH}=\frac{U_{TSH}}{U_{T}}\times 100\%\\ P_{F}=\frac{U_{F}}{U_{T}}\times 100\%\\ P_{FS}=\frac{U_{FS}}{U_{T}}\times 100\% \end{array}$$

where $P_{F-TSH}$, $P_{F}$, and $P_{FS}$ are the strain energy proportions of thickness–shear mode, flexural mode, and face–shear mode, respectively. Due to the small proportions of the coupled energies, they can be ignored in this work.

Modal identification can also help determine the dominant mode at the current frequency by further calculating the proportions of kinetic energies. The following equation is the expression for kinetic energy density.

(23) $$\bar{K}=\frac{1}{2}\sum_{m}\sum_{n}\rho B_{mn}{\dot{u}}_{j}^{\left(m\right)}{\dot{u}}_{j}^{\left(n\right)}=\frac{1}{2}\rho B_{00}{\dot{u}}_{2}^{\left(0\right)}{\dot{u}}_{2}^{\left(0\right)}+\frac{1}{2}\rho B_{00}{\dot{u}}_{3}^{\left(0\right)}{\dot{u}}_{3}^{\left(0\right)}+\frac{1}{2}\rho B_{11}{\dot{u}}_{1}^{\left(1\right)}{\dot{u}}_{1}^{\left(1\right)}$$

where the expression for the kinetic energy of each mode is

(24) $$\begin{array}{l} {\bar{K}}_{TSH}=\frac{1}{2}\rho B_{11}{\dot{u}}_{1}^{\left(1\right)}{\dot{u}}_{1}^{\left(1\right)}\\ {\bar{K}}_{F}=\frac{1}{2}\rho B_{00}{\dot{u}}_{2}^{\left(0\right)}{\dot{u}}_{2}^{\left(0\right)}\\ {\bar{K}}_{FS}=\frac{1}{2}\rho B_{00}{\dot{u}}_{3}^{\left(0\right)}{\dot{u}}_{3}^{\left(0\right)} \end{array}$$

Similar to the treatment of strain energy, we could also integrate the kinetic energy densities of different modes within one vibration period to obtain the kinetic energy of each mode, as shown in the following equation.

(25) $$K_{N}=\int_{-a}^{a}\int_{-c}^{c}\int_{0}^{T}{\bar{K}}_{N}dx_{1}dx_{3}dt,\;\;\;\left(N=TSH,FS,F,F-TSH,TSH-FS\right)$$

The total kinetic energy of all modes within one vibration period is given in Equation (26).

(26) $$K=\sum K_{N}=K_{TSH}+K_{F}+K_{FS}$$

Finally, the proportion of kinetic energy for each mode is given as follows.

(27) $$\begin{array}{l} P_{TSH}=\frac{K_{TSH}}{K}\times 100\%\\ P_{F}=\frac{K_{F}}{K}\times 100\%\\ P_{FS}=\frac{K_{FS}}{K}\times 100\% \end{array}$$

where $P_{TSH}$, $P_{F}$, and $P_{FS}$ are the kinetic energy proportions of thickness–shear mode, flexural mode, and face–shear modes, respectively.

## 3. Results and Discussion

In this section, we discuss the analysis results of modal identification based on the energy method. Due to the use of AT-cut quartz material in the model, their material properties in the following form can be found in the reference [30]. The density of quartz was 2649 kg/m^3^, and the material properties of AT-cut quartz is given below.

$$\left[\begin{array}{cccccc} 86.74 & -8.25 & 27.15 & -3.66 & 0 & 0\\ -8.25 & 129.77 & -7.42 & 5.7 & 0 & 0\\ 27.15 & -7.42 & 102.83 & 9.92 & 0 & 0\\ -3.66 & 5.7 & 9.92 & 38.61 & 0 & 0\\ 0 & 0 & 0 & 0 & 68.81 & 2.53\\ 0 & 0 & 0 & 0 & 2.53 & 29.01 \end{array}\right]\times 10^{9}N/m^{2}$$

Before beveling process, the geometric dimensions of the blank quartz plate were 1659 μm × 129 μm × 1311 μm in length, thickness, and width, respectively. After a period of beveling, a blank quartz plate can obtain a contour with varying thickness. For the convenience of calculation, we fitted the contour using a quadratic polynomial, which can be expressed as the following equation.

$$h(x_{1},x_{3})=\alpha_{1}x_{1}^{2}+\alpha_{2}x_{3}^{2}+\alpha_{3}x_{1}x_{3}+\alpha_{4}x_{1}+\alpha_{5}x_{3}+\alpha_{6}$$

where the parameters such as *α*_1_, *α*_2_, *α*_3_, *α*_4_, *α*_5_, and *α*_6_ were fitted through morphology measurements. It should be noted that they mathematically described the morphology and did not have direct physical interpretations related to the actual physical properties of the plate.

Next, we could substitute the above thickness function into the previous mechanical model and ultimately solve the governing equation through numerical method to obtain eigenvalues and eigenvectors. The beveling process usually requires several tens of hours of processing, so we measured some morphological parameters of the intermediate process and fitted them with the above polynomial to obtain the parameter values in the polynomial. Here, we measured four sets of thickness values at the beveling times of 10 H, 20 H, 30 H, and 40 H. The coefficients obtained after fitting are given as follows.

*α*_1_ = −14.00, *α*_2_ = −18.26, *α*_3_ = −0.07, *α*_4_ = 0.02, *α*_5_ = 0.02, *α*_6_ = 46.46 × 10^−6^

*α*_1_ = −19.40, *α*_2_ = −25.26, *α*_3_ = −0.05, *α*_4_ = 0.03, *α*_5_ = 0.04, *α*_6_ = 37.48 × 10^−6^

*α*_1_ = −12.57, *α*_2_ = −17.36, *α*_3_ = −0.11, *α*_4_ = 0.02, *α*_5_ = 0.02, *α*_6_ = 42.63 × 10^−6^

*α*_1_ = −24.78, *α*_2_ = −31.66, *α*_3_ = −0.10, *α*_4_ = 0.04, *α*_5_ = 0.04, *α*_6_ = 26.25 × 10^−6^

Then, we substituted the calculated modal displacement and frequency into the energy formula to obtain the energy of the plate, thereby further identifying the vibration modes in the variable thickness quartz plate. It is worth noting that this paper only conducted vibration modal identification on variable thickness quartz crystal plates. For modal identification of flat plates, readers can refer to the reference [29]. In the eigenvalue analysis of COMSOL, we set the first-order thickness–shear vibration frequency of an infinite plate equal to the maximum thickness value of the beveled plate as the reference point for eigenvalue solving, and we performed the frequency search and normalization near this value. Among them, the frequency used for normalization was the thickness–shear vibration frequency of an infinite plate, that is, $\sqrt{c_{66}/\rho }/4/b$. For validation, we also calculated the thickness–shear mode of the flat plate by using COMOSL for the beveling time is zero. The fundamental thickness–shear mode was 12.936 MHz, while it was 12.827 MHz using the equation for an infinite plate. The two calculation results were well validated. Then, we calculated 100 characteristic frequencies near the reference point and calculated the energy proportions of the corresponding modes for the first 20 frequencies. The different modal strain energy and kinetic energy proportions calculated at four different beveling time conditions are shown in Table 1, Table 2, Table 3 and Table 4.

Firstly, it can be observed from the four tables that the normalized frequency increases with the increase of beveling time. The first 20 normalized frequencies we selected all increased with the duration of the beveling. This indicates that as the time for beveling increased, the thickness of the quartz plate became thinner, resulting in an increase in the thickness–shear frequency. Secondly, it could be observed from the energy proportions of the three modes that when the beveling time was 10 H, the strain energy and kinetic energy occupied by the TSH mode were at a relatively small value, except for the case where the normalized frequency was 1.0215. Under this frequency condition, TSH mode was the dominant mode, and the energy proportions of the other two modes were less than 2%. In addition, under most frequency conditions, the energy proportion of the F mode was relatively high, and the FS mode also dominated in some cases, but this situation was less than that of the F mode. Therefore, from Table 1, it can be summarized that in this three-mode coupled vibration, both the F mode and the FS mode affected the TSH mode, but the F mode had a greater impact and was highly likely to become the main component of the spurious mode. The above conclusions can also be verified in Table 2 and Table 3. On the other hand, with the increase of the beveling time from Table 1 to Table 4, we could also find that when the TSH vibration mode became the dominant mode, the proportions of strain energy and kinetic energy slightly increased. This also verified the energy trapping effect from another perspective, that is, the beveling could improve the vibration performance of the resonator devices. Therefore, the energy proportion results from the four tables indicate that the energy method could more intuitively identify the dominant modes corresponding to each frequency, thereby better assisting engineers in modal identification and device design work.

**Figure 2:**
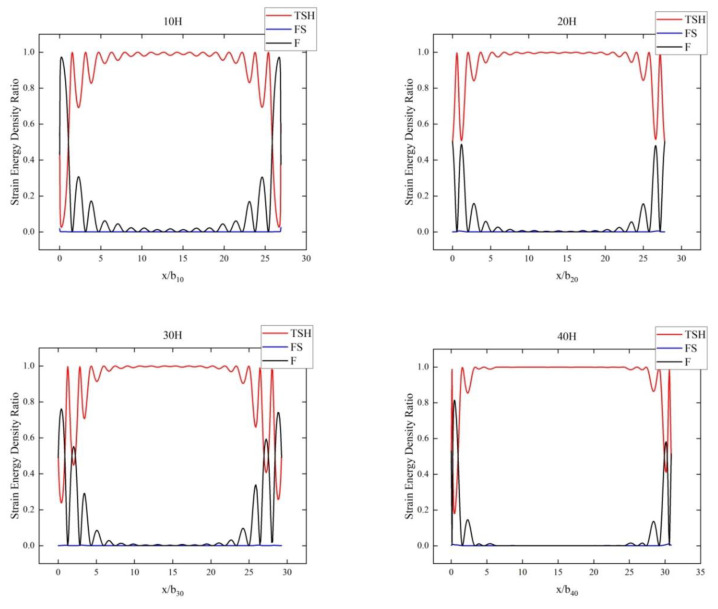
Strain energy density proportion of the three modes at the center line and different beveling times.

**Figure 3:**
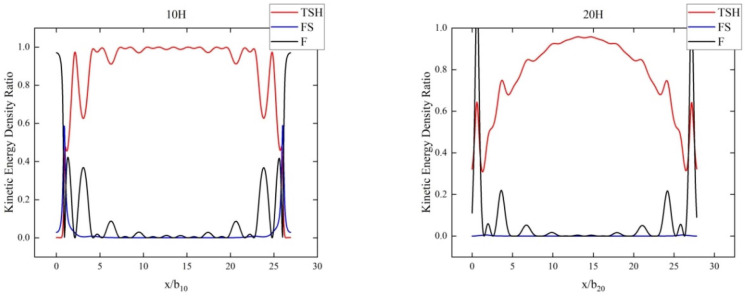

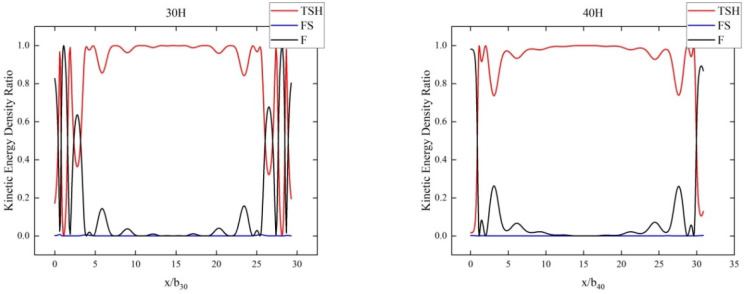
Kinetic energy density proportion of the three modes at the center line and different beveling times.

Secondly, Figure 2 and Figure 3, respectively, show the distribution of strain energy densities and kinetic energy densities along the centerline of the plate when TSH mode was the dominant mode at different beveling times. In these figures, the *x*-coordinate represents the long axis of the plate, and the *y*-coordinate represents the proportion of energy density. In Figure 2, we can observe that in the middle region of the plate, as the beveling time increased, the strain energy density of the TSH mode gradually stabilized and the oscillation disappeared, while the proportions of the other two modes gradually approached zero. At the edge of the plate, i.e., at both ends, two spurious modes could be found to occupy a larger energy density value. The dominant role of TSH mode was not strong at the edge of the board, and the energy proportions of spurious modes were relatively high, which had a significant impact. This oscillatory behavior indicates more complex interactions between modes at the edges, where energy was less concentrated and more susceptible to interference from other modes. Therefore, it can be clearly indicated from the proportion of strain energy density that the energy trapping effect became more significant with increasing beveling time, and the TSH operating mode and spurious mode were also easier to identify. Similarly, a pattern of types could also be observed from the kinetic energy density results in Figure 3. The TSH mode, as the operating mode, was mainly concentrated in the middle region of the plate, and this concentration phenomenon became more significant with increasing beveling time. Therefore, the energy density distribution curves could also help us identify the dominant mode very well.

## 4. Conclusions

In this work, we developed a vibration mode identification method for variable-thickness quartz plates based on the Mindlin plate theory combined with the energy method. We first derived the formulas for the strain energy and kinetic energy of the coupled three modes of the beveled quartz plate and identified the dominant mode by calculating the energy proportion of each mode within one vibration period. From the results, it could be found that when the energy proportion was maximum, the corresponding mode was the dominant mode at that frequency, and the rest were spurious modes. It was also found from the results that as the beveling time increased, the operating mode frequency of the plate increased, and the energy trapping effect became more significant. This energy-based modal identification method is more intuitive and scientific compared to traditional modal identification methods and is more advantageous in the field of modal identification of devices composed of anisotropic materials with variable thickness. By focusing on energy ratios and their implications rather than simply relying on frequency separation, we can provide a more nuanced understanding of the vibrational characteristics of the quartz plate. Therefore, the energy-based modal identification method developed in this paper can provide an important analytical tool for modal identification and sensing device optimization of quartz resonators with variable thickness.

## Figures and Tables

**Table 1:** The energy proportion of the three modes at the beveling time of 10 H.

No.	Normalized *f*	TSH (S/K%)	F (S/K%)	FS (S/K%)
1	0.9852	23.8/11.1	**75.4/87.7**	0.8/1.1
2	0.9877	19.8/8.3	**79.3/91.3**	0.9/0.4
3	0.9907	13.3/5.2	**84.7/93.9**	2/0.9
4	0.9955	5.4/2	**91.1/96.5**	3.5/1.4
5	1.0115	7/2.7	**89.8/96**	3.2/1.3
6	1.0125	24.7/13.1	**74.8/86.5**	0.4/0.4
7	1.0143	0.1/0	0.7/1.8	**99.3/98.2**
8	1.0170	17.5/7.2	**81.3/92.3**	1.2/0.6
9	1.0187	0.8/0.4	0.5/0.8	**98.7/98.8**
10	1.0205	0.1/0.1	0.7/1.7	**99.2/98.2**
11	**1.0215**	**98.3/98.3**	1.7/1.7	0.1/0.1
12	1.0229	14.1/5.6	**84.1/93.6**	1.8/0.8
13	1.0281	21.3/9.3	**78.1/90.3**	0.6/0.4
14	1.0292	28.7/19.8	**71.1/79.9**	0.3/0.3
15	1.0299	0.8/0.3	0.8/0.5	**98.4/99.2**
16	1.0309	8.5/3.3	**88.6/95.5**	2.9/1.2
17	1.0316	22.5/10.4	**76.9/89.2**	0.6/0.5
18	1.0329	26.2/14.8	**73.6/85**	0.2/0.2
19	1.0387	19.7/8.5	**79.5/91**	0.8/0.4
20	1.0392	0.2/0.1	0.6/1.5	**99.2/98.3**

**Table 2:** The energy proportion of the three modes at the beveling time of 20 H.

No.	Normalized *f*	TSH (S/K%)	F (S/K%)	FS (S/K%)
1	1.0090	0.5/1.4	1.4/2.1	**98.1/96.5**
2	1.0107	0.5/1.4	2.4/3.4	**97.1/95**
3	1.0129	3.9/10.5	**95/87**	1.1/2.5
4	1.0132	6.1/15.8	**93.2/82.6**	0.7/1.5
5	1.0136	7.1/18.3	**92.4/80.6**	0.5/1.1
6	1.0218	0/0	**98.2/95.4**	1.8/4.6
7	1.0230	0.1/0.2	**98.1/95.2**	1.8/4.6
8	1.0264	0.3/0.9	**98/94.7**	1.7/4.4
9	1.0282	10.7/23.6	**87.9/75.5**	1.4/1.9
10	**1.0304**	**99/99.3**	1/0.7	0/0
11	1.0322	0.7/1.9	**97.6/93.9**	1.7/4.2
12	1.0334	0.2/0.4	1.3/0.6	**98.4/99**
13	1.0335	8.7/21.4	**90.9/78**	0.3/0.6
14	1.0374	4.5/11.7	**94.6/86**	1/2.3
15	1.0387	8.4/21.1	**91.3/78.3**	0.3/0.6
16	1.0406	1.2/3.1	**97.3/92.9**	1.6/4
17	1.0417	9.4/22	**90.1/77.4**	0.5/0.6
18	1.0424	10.8/24.8	**85/72.9**	4.2/2.3
19	1.0451	7.9/20.9	**63.2/61.9**	28.9/17.2
20	1.0462	4.7/13.9	33.3/40.3	**62/45.8**

**Table 3:** The energy proportion of the three modes at the beveling time of 30 H.

No.	Normalized *f*	TSH (S/K%)	F (S/K%)	FS (S/K%)
1	0.9897	16.6/6.1	**82/93.3**	1.4/0.6
2	0.9936	1.5/0.5	2.9/1.8	**95.6/97.7**
3	0.9954	23/9.2	**76.6/90.5**	0.4/0.4
4	0.9962	19.2/7.2	**79.9/92.3**	0.9/0.4
5	0.9971	4.7/1.7	**91.6/96.9**	3.7/1.5
6	0.9975	0.9/0.4	1.8/1.7	**97.3/97.9**
7	1.0064	13.1/4.8	**84.9/94.3**	2/0.9
8	1.0081	24.1/11.7	**74.9/86.7**	1/1.6
9	1.0097	6.2/2.2	**90.4/96.4**	3.4/1.4
10	**1.0196**	**99.1/98.9**	0.8/1.1	0/0
11	1.0203	0.6/0.3	0.5/1.2	**98.9/98.5**
12	1.0243	21.3/8.2	**78.1/91.4**	0.5/0.3
13	1.0251	8/2.9	**89/95.9**	3/1.2
14	1.0265	20.3/7.7	**79.1/92**	0.7/0.3
15	1.0343	22.1/8.8	**77.5/90.8**	0.5/0.4
16	1.0352	13.9/5.2	**84.3/94**	1.8/0.8
17	1.0369	24.6/9.4	**74.6/89.4**	0.7/1.2
18	1.0410	18.9/7.2	**80.2/92.4**	0.9/1.4
19	1.0418	24/9.4	**74.3/87.5**	1.7/3.1
20	1.0426	1.2/0.4	1.9/1.4	**96.8/98.2**

**Table 4:** The energy proportion of the three modes at the beveling time of 40 H.

No.	Normalized *f*	TSH (S/K%)	F (S/K%)	FS (S/K%)
1	1.0064	1.6/0.5	3.9/2.7	**94.5/96.8**
2	1.0067	15.4/5.3	**78.8/87.1**	5.8/7.6
3	1.0077	0/0	**95/98.1**	5/1.9
4	1.0081	22.5/8.6	**75.7/88.3**	1.8/3.1
5	1.0083	11.2/3.9	**85.7/94.3**	3.1/1.8
6	1.0085	0.2/0.1	**94.8/98**	5/1.9
7	1.0095	20/6.7	**74.2/83.7**	5.8/9.6
8	1.0101	7.4/1.7	26.5/20	66/78.2
9	1.0111	21.9/8.1	**77/90.3**	1.1/1.6
10	1.0114	0.8/0.3	**94.5/97.9**	4.7/1.8
11	**1.0121**	**99.9/99.4**	0.1/0.6	0/0
12	1.0123	1.1/0.3	1.7/1.1	**97.2/98.6**
13	1.0154	25.8/8.5	**74/91.3**	0.2/0.2
14	1.0160	1.7/0.6	**93.8/97.6**	4.6/1.8
15	1.0217	23/8.6	**76.4/90.5**	0.6/0.9
16	1.0224	19.9/7.4	**79.2/92.2**	0.9/0.4
17	1.0225	6.3/2	**90.1/96.5**	3.6/1.5
18	1.0227	24.2/8.4	**75.4/91**	0.5/0.6
19	1.0262	23.2/8.4	**75.7/89.9**	1.1/1.7
20	1.0303	12/4.3	**85.5/94.6**	2.5/1.1

## Data Availability

The data presented in this study are available on request from the corresponding author.

## References

[1] Reynders E. (2012). System identification methods for (Operational) modal analysis: Review and comparison. Arch. Comput. Methods Eng..

[2] Wang C., Wang J.Y., Zhang T.S. (2017). Operational modal analysis for slow linear time-varying structures based on moving window second order blind identification. Signal Process..

[3] Fan C., Shi J., Zhao M., Yang J. (2015). Trapped thickness-shear modes in a contoured, partially electroded AT-cut quartz resonator. Eur. Phys. J.-Appl. Phys..

[4] Lee P., Wang J. Frequency-temperature relations of thickness-shear and flexural vibrations of contoured quartz resonators. Proceedings of the 1996 IEEE International Frequency Control Symposium.

[5] Zhao Z., Qian Z., Wang B., Yang J. (2015). Thickness-shear and thickness-twist modes in an AT-cut quartz acoustic wave filter. Ultrasonics.

[6] Sekimotoy H., Watanabe Y., Tanaka K., Nakazawa M. (2010). Two-dimensional analysis of coupled thickness-shear and flexural vibrations in rectangular AT-cut quartz resonators using a finite-element method. Electr. Commun. Jpn..

[7] Tiersten H.F., Smythe R.C. (1985). Coupled thickness-shear and thickness-twist vibrations of unelectroded AT-cut quartz plates. J. Acoust. Soc. Am..

[8] Zheng Y., Huang B., Wang J. (2021). Flexoelectric effect on thickness-shear vibration of a rectangular piezoelectric crystal plate. Mater. Res. Express.

[9] Sun Z., Zheng Y., Guo Y., Huang B. (2024). Size effect on the nonlinear thickness-shear vibration of an elliptical piezoelectric plate. J. Vib. Eng. Technol..

[10] Zhang Y., Han T. (2014). Effects of electrode configuration on vibration characteristics of quartz thickness-shear mode trapped-energy resonators. Ferroelectr. Lett..

[11] Apostolov A.V., Slavov S.H. (1982). Frequency spectrum and modes of vibration in circular, convex AT-cut bevelled—Design quartz resonators. Appl. Phys. A.

[12] Jeong H.W., Aoki T., Hatsuzawa T. (2004). Frequency responses of spherically contoured rectangular AT-cut quartz crystal resonators fabricated by fixed abrasive method. Int. J. Mach. Tools Manuf..

[13] Wang J., Yang J., Li J. (2007). Energy trapping of thickness-shear vibration modes of elastic plates with functionally graded materials. IEEE Trans. Ultrason. Ferroelectr. Freq. Control..

[14] Yang J., Xue H., Fang H., Hu Y., Wang J., Shen L. (2007). Effects of electrodes with varying thickness on energy trapping in thickness-shear quartz resonators. IEEE Trans. Ultrason. Ferroelectr. Freq. Control..

[15] Mindlin R.D., Forray M. (1954). Thickness-shear and flexural vibrations of contoured crystal plates. J. Appl. Phys..

[16] Yang J.S., Batra R.C. (1995). Thickness shear vibrations of a circular cylindrical piezoelectric shell. J. Acoust. Soc. Am..

[17] Wang J., Lee P.C.Y., Bailey D.H. (1999). Thickness-shear and flexural vibrations of linearly contoured crystal strips with multiprecision computation. Comput. Struct..

[18] Slavov S.H. (1987). Equivalent resonance radius of contoured AT-cut quartz resonators. Appl. Phys. A.

[19] Tiersten H.F., Lwo B.J., Dulmet B. (1996). Transversely varying thickness modes in trapped energy resonators with shallow and beveled contours. J. Appl. Phys..

[20] Tiersten H.F., Smythe R.C. (1979). An analysis of contoured crystal resonators operating in overtones of coupled thickness-shear and thickness-twist. J. Acoust. Soc. Am..

[21] Li P., Jin F., Yang J. (2012). Thickness-shear vibration of an AT-cut quartz resonator with a hyperbolic contour. IEEE Trans. Ultrason. Ferroelectr. Freq. Control..

[22] Li P., Jin F. (2017). The investigation of trapped thickness shear modes in a contoured AT-cut quartz plate using the power series expansion technique. J. Phys. D Appl. Phys..

[23] Wang W., Wu R., Wang J., Du J., Yang J. (2013). Thickness-shear modes of an elliptical, contoured AT-cut quartz resonator. IEEE Trans. Ultrason. Ferroelectr. Freq. Control..

[24] Wang J., Lee P.C.Y. The effect of cubically varying contours on the thickness-shear and flexural vibrations of quartz plates. Proceedings of the IEEE Ultrasonics Symposium. Proceedings.

[25] Lu F., Lee H.P., Lim S.P. (2005). Energy-trapping analysis for the bi-stepped mesa quartz crystal microbalance using the finite element method. Smart Mater. Struct..

[26] He H., Liu J., Yang J. (2011). Thickness-shear and thickness-twist vibrations of an AT-cut quartz mesa resonator. IEEE Trans. Ultrason. Ferroelectr. Freq. Control..

[27] Yong Y.K., Stewart J.T., Detaint J., Zarka A., Capelle N., Zheng Y. Thickness-shear mode shapes and mass-frequency influence surface of a circular and electroded AT-cut quartz resonator. Proceedings of the 45th Annual Symposium on Frequency Control.

[28] Wang J., Zhao W., Bian T. A fast analysis of vibrations of crystal plates for resonator design applications. Proceedings of the 2004 IEEE International Frequency Control Symposium and Exposition.

[29] Huang Q., Wu R.X., Wang L.H., Xie L.T., Du J.K., Ma T.F., Wang J. (2020). Identification of vibration modes of quartz crystal plates with proportion of strain and kinetic energies. Int. J. Acoust. Vib..

[30] Yang J. (2006). Analysis of Piezoelectric Devices.

